# Standard isometric contraction has higher reliability than maximum voluntary isometric contraction for normalizing electromyography during level walking among older adults with knee osteoarthritis

**DOI:** 10.3389/fbioe.2024.1276793

**Published:** 2024-02-16

**Authors:** Xiaoxue Zhu, Yaya Pang, Li Li, Wei Sun, Lijie Ding, Qipeng Song, Peixin Shen

**Affiliations:** ^1^ College of Sports and Health, Shandong Sport University, Jinan, China; ^2^ Department of Health Sciences and Kinesiology, Georgia Southern University, Statesboro, GA, United States

**Keywords:** EMG normalization, knee osteoarthritis, reliability, MVIC, gait

## Abstract

**Introduction:** Electromyography (EMG) normalization often relies on maximum voluntary isometric contraction (MVIC), which may not be suitable for knee osteoarthritis (KOA) patients due to difficulties in generating maximum joint torques caused by pain. This study aims to assess the reliability of standard isometric contraction (SIC) for EMG normalization in older adults with KOA, comparing it with MVIC.

**Methods:** We recruited thirty-five older adults with KOA and collected root mean square EMG amplitudes from seven muscles in the affected limb during level walking, SIC, and MVIC tests. EMG data during level walking were normalized using both SIC and MVIC methods. This process was repeated after at least 1 week. We calculated intra-class correlation coefficients (ICCs) with 95% confidence intervals to evaluate between- and within-day reliabilities.

**Results:** SIC tests showed higher between- (ICC: 0.75–0.86) and within-day (ICC: 0.84–0.95) ICCs across all seven muscles compared to MVIC tests. When normalized with SIC, all seven muscles exhibited higher between- (ICC: 0.67–0.85) and within-day (ICC: 0.88–0.99) ICCs compared to MVIC normalization.

**Conclusion:** This study suggests that SIC may offer superior movement consistency and reliability compared to MVIC for EMG normalization during level walking in older adults with KOA.

## 1 Introduction

Knee Osteoarthritis (KOA) is a chronic degenerative disease caused by the degeneration of articular cartilage through wear and tear (Lv et al., 2021), commonly occurring in older adults (Heidari, 2011), with clinical symptoms including pain, stiffness, and instability (Hsu and Siwiec, 2023). Knee muscle activation and its induced gait pattern were different in KOA patients compared to healthy controls, which may be a compensatory strategy to cope with KOA symptoms (Hubley-Kozey et al., 2006). Electromyography (EMG) is a biomedical signal that measures the electrical currents generated during muscle contractions that represent neuromuscular activity (Raez, Hussain, and Mohd-Yasin, 2006). EMG can be used to dynamically observe the degree and duration of muscle activation. Analysis of EMG during level walking among older adults with KOA helps to understand the changes in muscle activation and knee biomechanics (Schrijvers et al., 2021) and can be beneficial in the diagnosis, treatment, and rehabilitation of KOA (Sun et al., 2017; Kour, Gupta, and Arora, 2021).

Normalization influences the consistency and comparability of EMG among older adults with KOA (Kellis and Baltzopoulos, 1996; French et al., 2015). A normalization method should be reliable to ensure that EMG signals from the muscles are credible in identifying muscle dysfunction and evaluating treatment efficacy (Dankaerts et al., 2004a). The more reliable the SIC normalization method is, the more accurately researchers will be able to compare and analyze muscle activation among older adults with KOA, thus gaining insights into muscle activation patterns, and even the etiology of KOA, to develop precise rehabilitation programs.

Maximum voluntary isometric contraction (MVIC) is the most common approach for normalizing EMG (Merletti and Di Torino, 1999), but it may not be suitable for older adults with KOA. To obtain EMG during MVIC, participants need to maximize muscle contraction, and older adults with KOA may be unable or unwilling to produce maximal muscle contraction due to pain, stiffness, instability, muscle weakness, arthrogenous muscle inhibition, and other factors (Thomas et al., 2008). In addition, there is a risk of increasing blood pressure during MVIC, which may be harmful to older adults (Watanabe et al., 2022). When participants cannot perform MVIC due to pain or risk of injury (Besomi et al., 2020), traditional sub-MVIC provides an alternative that allows participants to contract their muscles at a certain percentage of MVIC. However, the percentage varies in the literature, like 30% (Yang and Winter 1983), 60% (Norasi, Koenig, and Mirka, 2022), or 75% (Doorenbosch, Joosten, and Harlaar, 2005). The percentage is difficult to determine for older adults with KOA, given the pain level and strength degradation vary between individuals and conditions. Standard isometric contraction (SIC) could be viewed as a unique form of sub-MIVC, in which participants remain in a standard position and isometrically contract their muscles to resist the body weight (Dankaerts et al., 2004b). It is called “standard” because the contraction force is relatively consistent and depends on body weight only. SIC differs from other calibration methods such as MVIC or traditional Sub-MVIC in that it requires participants to perform isometric contractions in a standard antigravity position rather than maximal or submaximal muscle contractions, thus avoiding the effects of factors such as pain and joint instability on the reproducibility of movements, and therefore has the potential to improve the movement or EMG consistency.

Therefore, this study has a two-fold objective: 1) to assess the between- and within-day reliabilities of EMG during SIC and MIVC tests among older adults with KOA; and 2) to assess the between- and within-day reliabilities of EMG during level walking normalized with SIC or MVIC among older adults with KOA. We hypothesized that: 1) among older adults with KOA, the between- and within-day ICCs of EMG are higher during SIC tests than in MVIC tests; and 2) compared with MIVC, the between- and within-day ICCs of EMG normalized with SIC are higher during level walking.

## 2 Methods

### 2.1 Participants

All participants were recruited from local communities by distributing flyers and providing presentations. Thirty-five participants voluntarily participated in this study. The inclusion criteria were: 60 years or older; diagnosed with unilateral KOA according to the clinical criteria of the American College of Rheumatology (Altman et al., 1986); and a Kellgren/Lawrence (K/L) grade of 2–4 as determined by an orthopedic specialist based on a patient’s x-rays (Kellgren and Lawrence, 1957). The exclusion criteria were: suffering from a neurological or neuromuscular disorder that affects the knee (other than the KOA); had a history of any lower extremity joint surgery or fractures in the past 3 months; reported chronic, disabling back, hip, ankle, or foot pain that affected their daily activities; used an assistive walking aid regularly; or had severe cognitive impairment according to Mini-mental State Examination (score<24 points). All participants gave their written informed consent. Human participation was approved by Institutional Review Boards in Shandong Sport University (2020018) and was in accordance with the Declaration of Helsinki.

### 2.2 Target muscles

The seven target muscles in this study were gluteus maximus (GM), semitendinosus (SD), rectus femoris (RF), vastus lateralis (VL), tibialis anterior (TA), gastrocnemius lateral (GL), and soleus (SOL) of the affected leg. These seven muscles were commonly measured in gait analysis (Burden, Trew, and Baltzopoulos, 2003; Hubley-Kozey et al., 2009; Elsais et al., 2020), including one- and two-joint hip extensor (GM and SD), two-joint hip flexor (RF), one- and two-joint knee extensor (VL and RF), two-joint knee flexor (SD and GL), one- and two-joint plantar flexor (SOL and GL), and one-joint dorsiflexor (TA).

### 2.3 Protocols

The tests were conducted at the Biomechanics Laboratory of Shandong Sport University. The enrolled participants visited the laboratory twice for two experimental sessions separated by at least 1 week. The participants’ demographics such as age, height, body mass, body mass index, and pain score were recorded. The pain score of the affected leg was assessed by five pain items of the Western Ontario McMaster Universities Osteoarthritis Index (WOMAC) (Bellamy et al., 1988). In each item, 0 points represented “no pain”, whereas 10 points represented “the worst pain possible”. Higher scores indicate more severe pain.

**FIGURE 1:**
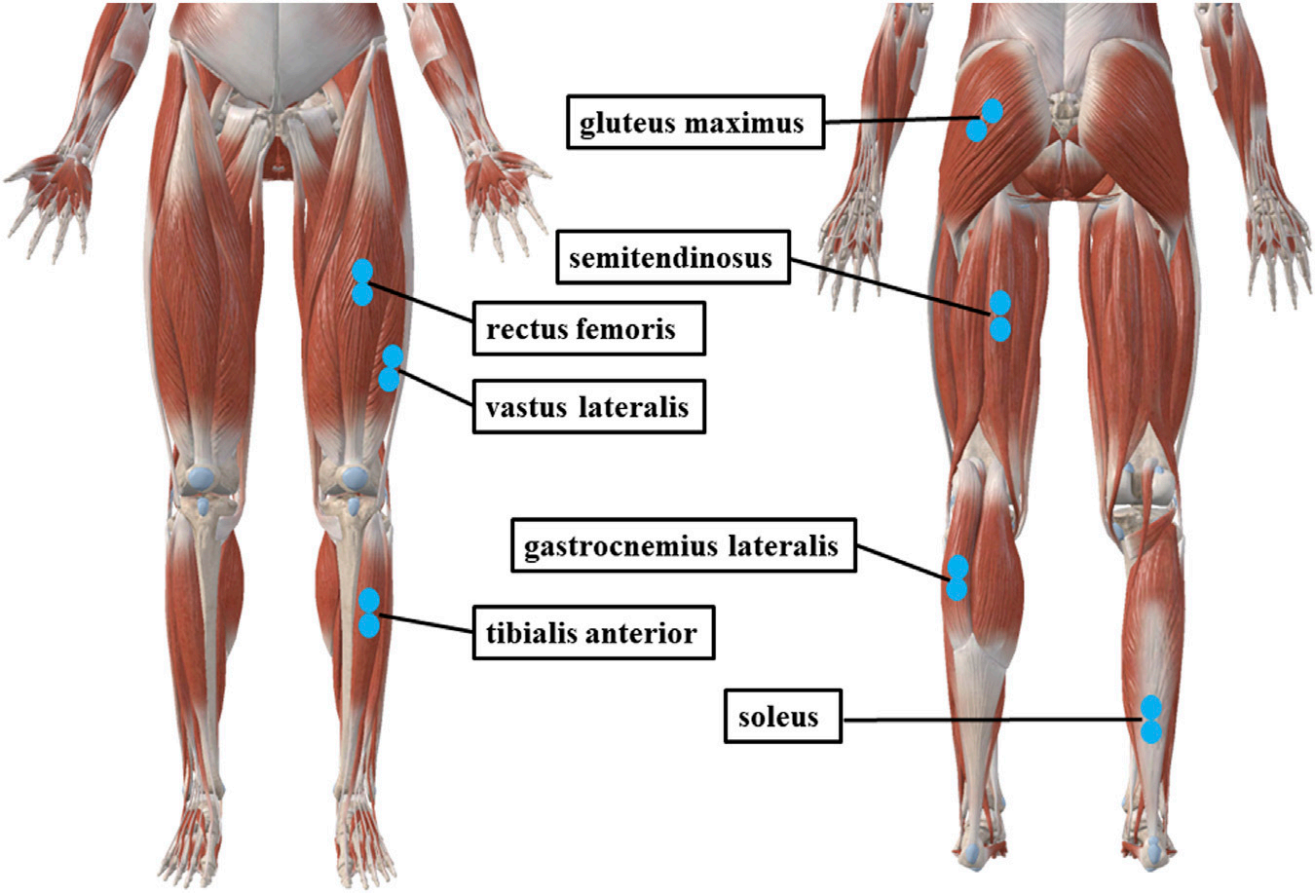
Electrode placement diagram. This image was taken from 3D body and used with permission.

EMG was collected during each session from level walking, SIC, and MVIC tests in a fixed order. EMG was recorded from the GM, SD, RF, VL, TA, GL, and SOL using a 16-channel EMG system at 2000 Hz (Ultium 8801, Noraxon Inc, United States of America). Before applying the electrodes, the skin was lightly shaved and cleaned with 70% alcohol wipes. According to the SENIAM recommendations (Hermens et al., 1999), Ag/AgCl surface electrodes (10 mm diameter, 20 mm inter-electrode distance) were placed over the seven muscles. The electrodes were placed as close to the center of the muscle belly as possible in view of the run of muscle fibers while avoiding the motor point (Warfel, 1985; De Luca and Carlo, 1997) (Figure 1). Forty-three reflective markers were placed according to the full-body Plug-in-Gait model (Paterson et al., 2017). Kinematic parameters were measured by using a 12-camera motion analysis system at 100 Hz (Vicon, Oxford Metrics Ltd., United Kingdom).

#### 2.3.1 Level walking test

Each participant walked at a self-selected speed on a 10 m walkway, and a minimum of six gait cycles were included in each test for further analysis (Shiavi et al., 1998). EMG from the participants’ affected legs was collected. Participants with KOA had different walking speeds, so self-selected speeds were chosen to increase the consistency of the EMG tests (Kadaba et al., 1989). The gait cycle was defined as the time between two consecutive foot-contact instances of the same foot, and the instance of foot-contact was calculated by the kinematic variables (Zeni et al., 2008).

#### 2.3.2 SIC test

**FIGURE 2:**
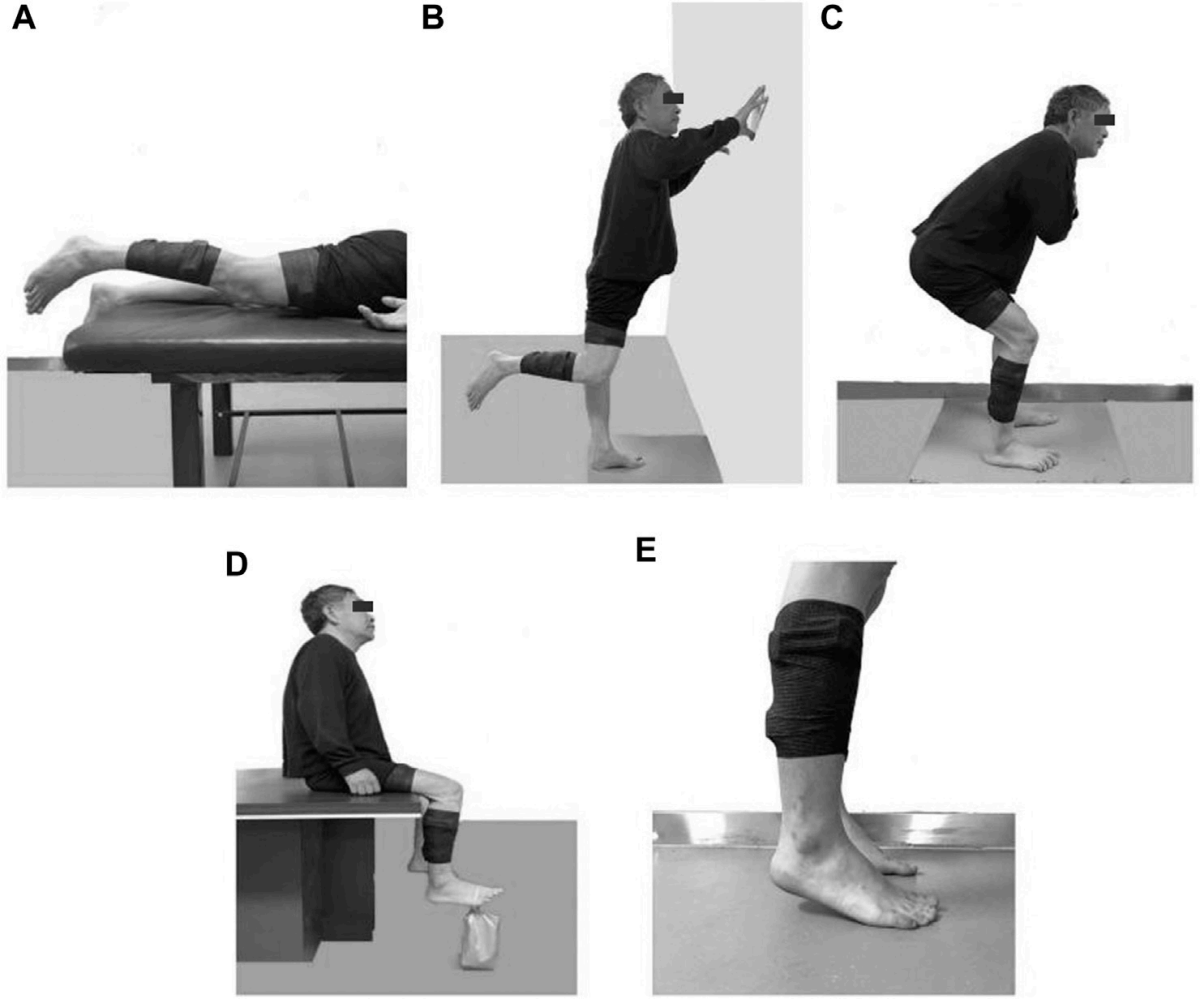
Illustrations of SIC tests for different muscles **(A)** Gluteus maximus, **(B)** Semitendinosus, **(C)** Rectus femoris and Vastus lateralis, **(D)** Tibialis anterior, **(E)** Gastrocnemius lateral and Soleus.

Participants extended their affected hips until the knees were 3 cm above the bed’s surface while lying prone during the GM tests (Figure 2A), stood on their healthy legs, and bent their affected knees to 90° during the SD tests (Figure 2B), squatted while kept their backs straight and flexed their trunks to 45° and knees to 120° during the RF and VL tests (Figure 2C), sat on a chair with their hips and knees flexed to 90° during the TA tests, dorsiflexed their affected ankles while strapping a sandbag weighing approximately 5% of the body weight to the metatarsophalangeal joint during the TA test (Figure 2D), stood on their forefeet with their heels raised to about 2 cm above the ground during the GL and SOL tests (Figure 2E). During RF and VL, and GL and SOL tests, the participants were verbally reminded to bear weight evenly on both lower limbs. Each test lasted for at least 5 s and was repeated thrice. Participants rested for 20 s between successive repetitions, or longer if needed.

#### 2.3.3 MVIC test

During the MVIC test, the participant’s body and joint positions were as follows: prone position with hips extended at 20° during the GM test; prone position with knees flexed at 30° during the SD test; seated position with knees flexed at 90° during the RF and VL tests; supine position with ankles plantarflexed at 90° during the GL and SOL tests (Konrad, 2005). Participants were instructed to maintain a standardized position against maximal manual resistance and were verbally encouraged to contract with the maximum effort and hold for at least 5 s. Each test was repeated thrice, and participants were allowed to rest for 120 s between successive repetitions (De Luca and Carlo, 1997).

### 2.4 Data reduction

**FIGURE 3:**
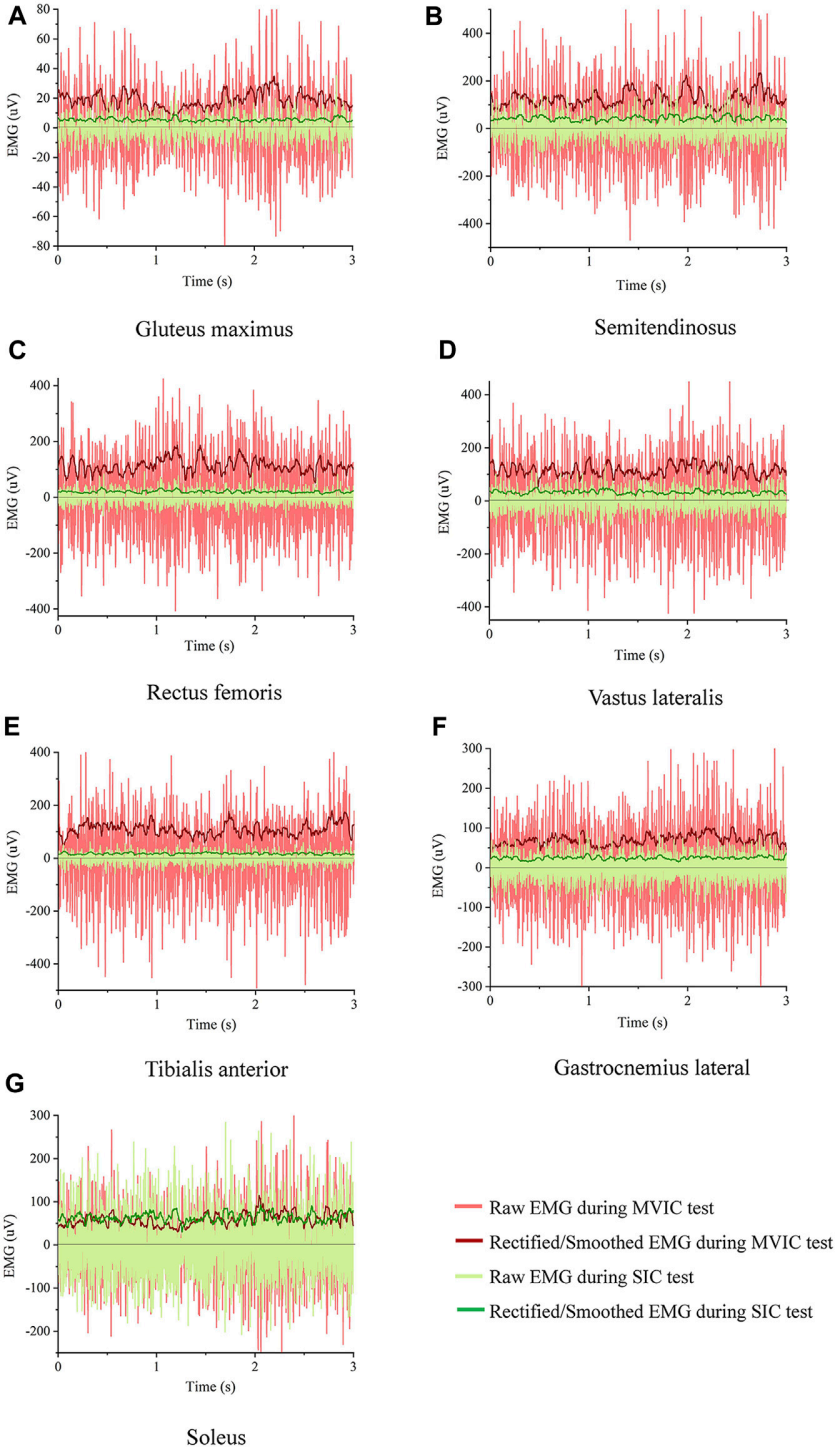
Raw and rectified/smoothed EMG in all seven muscles from an exemplary participant during the SIC and MVIC tests **(A)** Gluteus maximus, **(B)** Semitendinosus, **(C)** Rectus femoris, **(D)** Vastus lateralis, **(E)** Tibialis anterior, **(F)** Gastrocnemius lateral, **(G)** Soleus.

**FIGURE 4:**
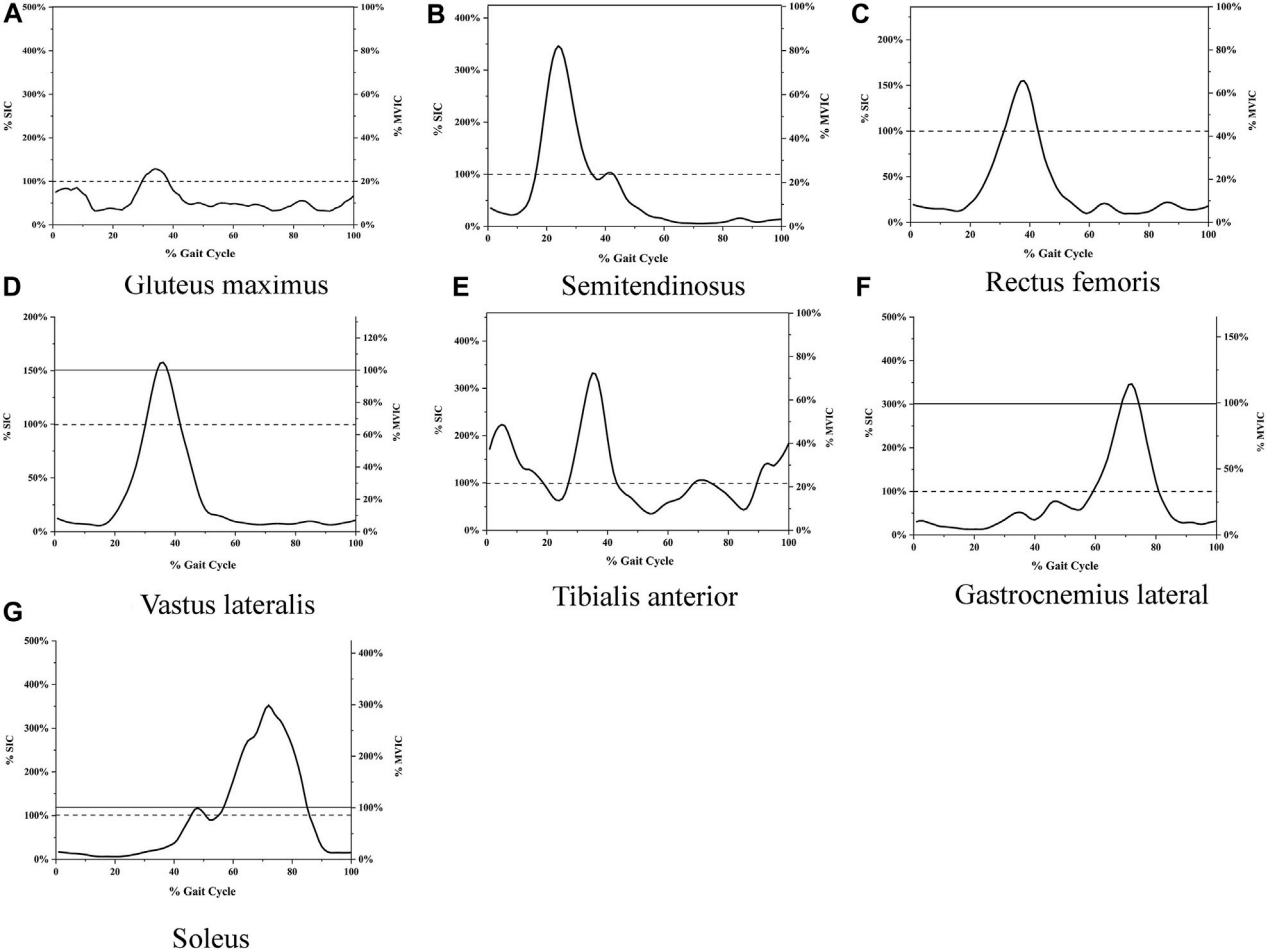
**(A)** EMG during level walking normalized with both SIC and MVIC Gluteus maximus, **(B)** Semitendinosus, **(C)** Rectus femoris, **(D)** Vastus lateralis, **(E)** Tibialis anterior, **(F)** Gastrocnemius lateral, **(G)** Soleus. The horizontal axis represents the percentage of the gait cycle, the left vertical axis represents EMG amplitudes normalized with SIC, and the right vertical axis represents EMG amplitudes normalized with MVIC. The curve represents the average root mean square of six gait cycles. The solid line indicates 100% of MVIC; the dashed line represents 100% of SIC.

The root mean square of EMG amplitudes from the level walking, SIC, and MVIC tests were processed using MR3 software (version 3.14, Noraxon Inc, United States). Visual inspection ruled out EMG noise due to poor electrode contact or skin movement artifacts during level walking. Raw EMG was band-pass filtered between 20 and 500 Hz (Tabard-Fougère et al., 2018). Then, the filtered data is rectified and filtered, with RMS calculated using a moving 50 m time window. (Albertus-Kajee et al., 2011), from six gait cycles during level walking. The EMG from the first and last seconds of each trial was discarded, and the mean EMG of the remaining 3s during SIC and MVIC tests was used as the reference amplitudes (Ha et al., 2013). The raw and rectified/smoothed EMG in all seven muscles during the SIC and MVIC tests is shown in Figure 3. The EMG of the lower limbs during level walking was normalized using SIC and MVIC (Figure 4). The mean values of normalized EMG from the six gait cycles were used to calculate the ICCs. The normalized EMG was calculated by dividing the gait EMG by the reference amplitudes. The between-day reliabilities were calculated using the normalized EMG during level walking of the first and second visits, while the within-day reliabilities were calculated using the normalized EMG among six repeated measurements of the first visit.

### 2.5 Data analysis

Statistics were done using SPSS software (Version 22; SPSS Inc, United States). Intra-class correlation coefficient (ICC) and 95% confidence interval (95% CI) were calculated to assess test-retest reliabilities (Koo and Mae, 2016). The ICCs were calculated by a two-way mixed model (Müller and Büttner, 1994). The thresholds for ICCs were little (0–0.25), low (0.26–0.49), moderate (0.50–0.69), high (0.70–0.89), and very high (0.90–1) (Wedege et al., 2017).

The ICCs were calculated using the following formula:

$$ICC\left(3,k\right)=\left(MSB-MSE\right)/\left(MSB+\left(MSR-MSE\right)/n\right)$$

Where MSB = between-subjects mean square; MSE = residual mean square; MSR = row mean square, indicating the mean square for raters; n = number of participants; k = number of raters.

## 3 Results

The demographics of the participants are shown in Table 1. Mean and standard deviation of age, height, mass, body mass index, Kellgren/Lawrence scale, and pain score were reported.

**TABLE 1:** Demographic characteristics of participants (n = 35).

Variable	Visit1	Visit2
Age (y)	65.00 ± 4.13	65.00 ± 4.13
Height (cm)	160.92 ± 8.16	160.92 ± 8.16
Mass (kg)	69.14 ± 9.41	69.28 ± 9.27
BMI (kg/m^2^)	26.69 ± 3.06	26.75 ± 3.00
K/L	12 II, 20 III, 3 IV	12 II, 20 III, 3 IV
WOMAC-pain	10.11 ± 3.40 (5)	10.29 ± 3.38 (5)

BMI: body mass index; K/L: Kellgren/Lawrence scale; WOMAC: Western Ontario and McMaster Universities Osteoarthritis Index.

The between-day ICCs of EMG during the SIC and MVIC tests are shown in Table 2. They were high in all seven muscles during the SIC tests, high in GM and SD, and moderate in RF, VL, TA, GL, and SOL during the MVIC tests. Compared with MVIC, the SIC had higher between-day ICCs in all seven muscles.

**TABLE 2:** Between-day reliabilities of EMG during the SIC and MVIC tests (n = 35).

Target muscles	SIC	MVIC
	ICC (95% CI)	ICC (95% CI)
Gluteus maximus	0.84 (0.70–0.91)	0.79 (0.62–0.89)
Semitendinosus	0.829 (0.689–0.91)	0.77 (0.57–0.88)
Rectus femoris	0.80 (0.63–0.89)	0.53 (0.24–0.73)
Vastus lateralis	0.80 (0.63–0.89)	0.65 (0.41–0.81)
Tibialis anterior	0.80 (0.63–0.89)	0.63 (0.38–0.79)
Gastrocnemius lateral	0.86 (0.75–0.93)	0.69 (0.47–0.83)
Soleus	0.75 (0.56–0.87)	0.68 (0.44–0.82)

The form of ICC, used employs a two-way mixed model and the average measures for evaluation. EMG: electromyography; SIC: standard isometric contraction; MVIC: maximum voluntary isometric contraction; CI: confidence intervals; ICC: intraclass correlation coefficient.

The within-day ICCs of EMG during the SIC and MVIC tests are shown in Table 3. They were high to very high in all seven muscles during the SIC tests, and high in seven muscles during the MVIC tests. Compared with MVIC, the SIC had higher within-day ICCs in all seven muscles.

**TABLE 3:** Within-day reliabilities of EMG during the SIC and MVIC tests (n = 35).

Target muscles	SIC	MVIC
	ICC (95% CI)	ICC (95% CI)
Gluteus maximus	0.94 (0.89–0.97)	0.84 (0.74–0.91)
Semitendinosus	0.89 (0.81–0.94)	0.75 (0.61–0.85)
Rectus femoris	0.89 (0.82–0.94)	0.85 (0.76–0.92)
Vastus lateralis	0.84 (0.74–0.91)	0.83 (0.73–0.91)
Tibialis anterior	0.92 (0.86–0.95)	0.80 (0.68–0.88)
Gastrocnemius lateral	0.90 (0.83–0.94)	0.89 (0.81–0.94)
Soleus	0.95 (0.92–0.98)	0.87 (0.77–0.93)

The form of ICC, used employs a two-way mixed model and the average measures for evaluation. EMG: electromyography; SIC: standard isometric contraction; MVIC: maximum voluntary isometric contraction; CI: confidence intervals; ICC: intraclass correlation coefficient.

The between-day ICCs of EMG amplitudes during level walking normalized with SIC and MVIC are shown in Table 4. They were high in RF, VL, TA, GL, and SOL and moderate in GM and SD using SIC normalization, whereas moderate in GM, SD, VL, TA, GL, and SOL and low in RF using MVIC normalization. Compared with MVIC, the between-day ICCs during level walking were higher in all seven muscles.

**TABLE 4:** Between-day reliabilities of EMG during level walking normalized with SIC or MVIC (n = 35).

Target muscles	SIC	MVIC
	ICC (95% CI)	ICC (95% CI)
Gluteus maximus	0.67 (0.43–0.82)	0.64 (0.39–0.80)
Semitendinosus	0.67 (0.35–0.83)	0.65 (0.41–0.81)
Rectus femoris	0.85 (0.72–0.92)	0.47 (0.16–0.69)
Vastus lateralis	0.79 (0.63–0.89)	0.61 (0.35–0.79)
Tibialis anterior	0.71 (0.45–0.85)	0.60 (0.34–0.78)
Gastrocnemius latera	0.74 (0.55–0.86)	0.55 (0.27–0.74)
Soleus	0.74 (0.54–0.86)	0.53 (0.25–0.74)

The form of ICC, used employs a two-way mixed model and the average measures for evaluation. EMG: electromyography; SIC: standard isometric contraction; MVIC: maximum voluntary isometric contraction; CI: confidence intervals; ICC: intraclass correlation coefficient.

The within-day ICCs of EMGs during level walking normalized with SIC and MVIC are shown in Table 5. They were high to very high in all seven muscles using SIC or MVIC normalization, and high in all seven muscles using MVIC normalization. Compared with MVIC, the within-day ICCs during level walking were higher in all seven muscles when normalized with SIC.

**TABLE 5:** Within-day reliabilities of EMG during level walking normalized with SIC or MVIC (n = 35).

Target muscles	SIC	MVIC
	ICC (95% CI)	ICC (95% CI)
Gluteus maximus	0.98 (0.93–0.99)	0.89 (0.87–0.89)
Semitendinosus	0.88 (0.61–0.95)	0.75 (0.55–0.94)
Rectus femoris	0.92 (0.74–0.97)	0.82 (0.74–0.87)
Vastus lateralis	0.97 (0.85–0.99)	0.87 (0.78–0.89)
Tibialis anterior	0.96 (0.86–0.99)	0.82 (0.70–0.97)
Gastrocnemius lateral	0.99 (0.95–1.00)	0.89 (0.88–0.90)
Soleus	0.94 (0.75–0.98)	0.88 (0.83–0.89)

The form of ICC, used employs a two-way mixed model and the average measures for evaluation. EMG: electromyography; SIC: standard isometric contraction; MVIC: maximum voluntary isometric contraction; CI: confidence intervals; ICC: intraclass correlation coefficient.

The EMG amplitudes normalized with SIC and MVIC in older adults with KOA during level walking are shown in Figure 4.

## 4 Discussion

This study aimed to assess the between- and within-day reliabilities of EMG during the SIC and MIVC tests, and level walking normalized with SIC and MVIC. The results support our two hypotheses, compared with MVIC, the SIC tests had higher between- and within-day ICCs; during level walking, the between- and within-day ICCs were higher when normalized with SIC than normalized with MVIC.

The outcomes indicated that the between-day reliabilities of EMG during the SIC tests were high in all seven muscles. A previous study supported us by reporting that the reliabilities were high in GM, RF, SD, VL, and TA during isoMMT3 tests, similar to the SIC we proposed, both of which require the target muscles to maintain isometric contraction against body weight (Tabard-Fougère et al., 2018). Besides, to obtain reliable EMG in GL and SOL, a standing with heel-rise movement was adopted, which was reliable among individuals with and without plantar flexion weakness (Yocum et al., 2010). Moreover, the outcomes indicated that the between-day reliabilities of EMG during the MVIC tests were moderate to high, and previous studies partly supported our observations. High between-day reliabilities in GM, RF, SD, and TA during the MVIC tests were obtained in a previous study (Tabard-Fougère et al., 2018). Besides, we detected moderate reliabilities in TA, RF, VL, GL, and SOL. These findings support our assumptions that the poor motivation due to pain among older adults with KOA during the MVIC tests would lead to inconsistency in developing maximum isometric contraction torques. This viewpoint was supported by a previous report, which observed poor between-day reliabilities in back muscles during MVIC tests among patients with low back pain (Lariviere et al., 2002). In addition, altered neuromechanics in patients with KOA, such as muscle weakness and joint muscle inhibition, may cause changes in joint loading patterns and cartilage response to joint loading, which affects the maximum isometric contraction torques, leading to poor between-day reliability (Seeley et al., 2022).

The higher retest reliability of the SIC compared to the MVIC may be attributed to two main reasons. Firstly, patients with KOA typically experience symptoms of joint instability and pain (Bertini and Làdavas, 2021; Hsu and Siwiec, 2023), which can significantly affect their motivation level to participate in MVIC tests (O’Sullivan et al., 2002). This leads to poorer consistency in the performance of MVIC tests, resulting in lower reliability. In contrast, SIC tests are less influenced by the motivation level. Testers only need to ensure that standard testing actions are maintained throughout the test to ensure consistency, thereby improving the retest reliability of SIC. Secondly, MVIC tests require participants to perform isometric contractions against maximal resistance, whereas SIC tests involve isometric contractions against gravity. Studies have shown that appropriate loading can maximally activate lower limb muscles, thereby enhancing the stability of the action. This increased action stability contributes to the consistency of surface electromyographic results across multiple measurements. Conversely, excessive loading can reduce action stability, leading to decreased action consistency and lower retest reliability.

The outcomes indicated that the within-day reliabilities of SIC and MVIC tests were high to very high in all seven muscles, and a previous study supported us by pointing out that both of them have good to excellent within-day reliabilities (Norcross et al., 2010). Compared with MVIC, the SIC tests had higher between- and within-day ICCs in all seven muscles. One possible reason is that the consistency of the EMG amplitude over a single trial is better in the SIC test than in the MVIC test, supported by the fact that the SIC-like isoMMT3 test shows better within-trial consistency (Tabard-Fougère et al., 2018).

The outcomes indicated that the between- and within-day reliabilities of EMG during level walking are higher when normalized by SIC compared with MVIC. Dankaerts and others supported our findings by reporting better reliabilities during sub-MVIC tests among participants with and without chronic low-back pain compared with MVIC tests (Dankaerts et al., 2004a). One possible reason was that SIC may have better movement consistency. During SIC tests, body posture can be adjusted in time to ensure consistency of movement. In contrast, the consistency of MVIC movements could be affected by the level of motivation (Ettinger et al., 2016), which is difficult to control and monitor during the tests (Beimborn and Morrissey, 1988; O’Sullivan et al., 2002). The target muscles were sub-maximally activated during the SIC tests, in which the consistency of movements may be less affected by pain among older adults with KOA. A previous study supported our viewpoint by pointing out that pain reduces maximal muscle activation but does not influence sub-maximal muscle activation among patients with musculoskeletal pain (Lund et al., 1991). The pain would diminish the motivation of older adults with KOA to develop maximum torques during the MVIC tests (Thomas et al., 2008). Compared with MVIC, the between-day reliabilities of EMG during level walking were statistically higher in RF using SIC normalization. During the RF test of MVIC, the participants were asked to extend their knee against maximum resistance, which is considered to most likely to cause knee pain (Lluch et al., 2018).

Besides the higher reliability, SIC has other advantages. Our results show that the reliability of the SIC test with a 20s rest period is higher than the reliability of the MVIC test with a 120s rest period, suggesting that the SIC tests have shorter periods and reduce the likelihood of fatigue. SIC may also apply to patients with pain or other musculoskeletal disorders (Wang et al., 2023), who may not be able to perform MVIC, and even if they could, they may have a higher risk of injury during the tests (Besomi et al., 2020).

This study has limitations. 1. Better reliability does not mean that SIC is more appropriate for comparing participant groups during gait (e.g., KOA *versus* healthy controls). SIC is appropriate to be used to compare within-participant EMG (e.g., pre- and post-session interventions), but it should be avoided when comparing EMG between groups of participants; 2. Interpretation with respect to physiology/mechanism is difficult because the reference value is not relative to the maximum capacity of the muscle; 3. Both MVIC and SIC were isometric contractions, they have different muscle fiber lengths and contraction types than dynamic-level walking. During level walking, muscle fiber length is constantly changing and therefore muscle activity is complex and variable, whereas in SIC testing, muscle fiber length remains constant and muscle activity is consistent (Vigotsky et al., 2017). Future studies are encouraged to compare the reliabilities between the SIC we proposed and functional dynamic normalization methods (Ball and Scurr, 2013); 4. Only two trials were conducted for each SIC or MVIC test, more trials were recommended in the future to reduce the variability of the data.

## 5 Conclusion

Compared with the MVIC, the SIC may have better movement consistency and be more reliable for normalizing EMG during level walking among older adults with KOA.

## Data Availability

The raw data supporting the conclusions of this article will be made available by the authors, without undue reservation.

## References

[B1] Albertus-Kajee Y. Tucker R. Derman W. Lamberts R. P. Lambert M. I. (2011). 'Alternative methods of normalising EMG during running. J. Electromyogr. Kinesiol 21, 579–586. 10.1016/j.jelekin.2011.03.009 21531148

[B2] Altman R. Asch E. Bloch D. Bole G. Borenstein D. Brandt K. (1986). Development of criteria for the classification and reporting of osteoarthritis: classification of osteoarthritis of the knee. Arthritis Rheum. 29, 1039–1049. 10.1002/art.1780290816 3741515

[B3] Ball N. Scurr J. (2013). 'Electromyography normalization methods for high-velocity muscle actions: review and recommendations. J. Appl. Biomech. 29, 600–608. 10.1123/jab.29.5.600 23270917

[B4] Beimborn D. S. Morrissey M. C. (1988). 'A review of the literature related to trunk muscle performance. Spine (Phila Pa 1976) 13, 655–660. 10.1097/00007632-198813060-00010 3051439

[B5] Bellamy N. Buchanan W. W. Goldsmith C. H. Campbell J. Stitt L. W. (1988). Validation study of WOMAC: a health status instrument for measuring clinically important patient relevant outcomes to antirheumatic drug therapy in patients with osteoarthritis of the hip or knee. J. Rheumatol. 15 (12), 1833–1840. 3068365

[B6] Bertini C. Làdavas E. (2021). 'Fear-related signals are prioritised in visual, somatosensory and spatial systems. Neuropsychologia 150, 107698. 10.1016/j.neuropsychologia.2020.107698 33253690

[B7] Besomi M. Hodges P. W. Clancy E. A. Van Dieën J. Hug F. Lowery M. (2020). 'Consensus for experimental design in electromyography (CEDE) project: amplitude normalization matrix. J. Electromyogr. Kinesiol 53, 102438. 10.1016/j.jelekin.2020.102438 32569878

[B8] Burden A. M. Trew M. Baltzopoulos V. (2003). 'Normalisation of gait EMGs: a re-examination. J. Electromyogr. Kinesiol 13, 519–532. 10.1016/s1050-6411(03)00082-8 14573367

[B9] Dankaerts W. O'Sullivan P. B. Burnett A. F. Straker L. M. Danneels L. A. (2004a). 'Reliability of EMG measurements for trunk muscles during maximal and sub-maximal voluntary isometric contractions in healthy controls and CLBP patients. J. Electromyogr. Kinesiol 14, 333–342. 10.1016/j.jelekin.2003.07.001 15094147

[B10] Dankaerts W. Peter B. O. ’S. Angus F. B. Leon M. S. Lieven A. D. (2004b). 'Reliability of EMG measurements for trunk muscles during maximal and sub-maximal voluntary isometric contractions in healthy controls and CLBP patients. J. Electromyogr. Kinesiol. 14, 333–342. 10.1016/j.jelekin.2003.07.001 15094147

[B11] De Luca Carlo J. (1997). 'The use of surface electromyography in biomechanics. J. Appl. biomechanics 13, 135–163. 10.1123/jab.13.2.135

[B12] Doorenbosch C. A. Joosten A. Harlaar J. (2005). 'Calibration of EMG to force for knee muscles is applicable with submaximal voluntary contractions. J. Electromyogr. Kinesiol 15, 429–435. 10.1016/j.jelekin.2004.11.004 15811613

[B13] Elsais W. M. Preece S. J. Jones R. K. Lee H. (2020). Between-day repeatability of lower limb EMG measurement during running and walking. J. Electromyogr. Kinesiol. 55, 102473. 10.1016/j.jelekin.2020.102473 33002858

[B14] Ettinger L. Weiss J. Shapiro M. Karduna A. (2016). Normalization to maximal voluntary contraction is influenced by subacromial pain. J. Appl. biomechanics 32, 433–440. 10.1123/jab.2015-0185 27115101

[B15] French H. P. Huang X. Cummiskey A. Meldrum D. Malone A. (2015). 'Normalisation method can affect gluteus medius electromyography results during weight bearing exercises in people with hip osteoarthritis (OA): a case control study. Gait posture 41, 470–475. 10.1016/j.gaitpost.2014.11.011 25600175

[B16] Ha S.-min Cynn H.-seock Kwon O.-yun Park K.-nam Kim G.-mo (2013). 'A reliability of electromyographic normalization methods for the infraspinatus muscle in healthy subjects. J. Hum. Kinet. 36, 69–76. 10.2478/hukin-2013-0007 23717356 PMC3661896

[B17] Heidari B. (2011). Knee osteoarthritis prevalence, risk factors, pathogenesis and features: Part I. Casp. J. Intern Med. 2 (2), 205–212. PMC3766936 24024017

[B18] Hermens Hj Freriks B. Merletti R. Hägg Gg Stegeman D. F. Blok J. H. 1999. "SENIAM 8: European recommendations for surface electromyography." In.

[B19] Hsu H. Siwiec R. M. (2023). Knee osteoarthritis. St. Petersburg, Florida, United States: StatPearls Publishing. 29939661

[B20] Hubley-Kozey C. L. Deluzio K. J. Landry S. C. McNutt J. S. Stanish W. D. (2006). 'Neuromuscular alterations during walking in persons with moderate knee osteoarthritis. J. Electromyogr. Kinesiol 16, 365–378. 10.1016/j.jelekin.2005.07.014 16213159

[B21] Hubley-Kozey Cheryl L. Hill N. A. Derek J. Dunbar M. J. Stanish W. D. (2009). 'Co-activation differences in lower limb muscles between asymptomatic controls and those with varying degrees of knee osteoarthritis during walking. Clin. Biomech. 24, 407–414. 10.1016/j.clinbiomech.2009.02.005 19303179

[B22] Kadaba M. P. Hk Ramakrishnan M. E. W. Gainey J. Gorton G. Cochran G. V. B. (1989). 'Repeatability of kinematic, kinetic, and electromyographic data in normal adult gait. J. Orthop. Res. 7, 849–860. 10.1002/jor.1100070611 2795325

[B23] Kellgren J. H. Lawrence J. S. (1957). 'Radiological assessment of osteo-arthrosis. Ann. rheumatic Dis. 16, 494–502. 10.1136/ard.16.4.494 PMC1006995 13498604

[B24] Kellis E. Baltzopoulos V. (1996). 'The effects of normalization method on antagonistic activity patterns during eccentric and concentric isokinetic knee extension and flexion. J. Electromyogr. Kinesiol. 6, 235–245. 10.1016/s1050-6411(96)00012-0 20719680

[B25] Konrad P. (2005). The abc of emg. A Pract. Introd. Kinesiol. Electromyogr. 1, 30–35.

[B26] Koo T. K. Mae Y. (2016). 'A guideline of selecting and reporting intraclass correlation coefficients for reliability research. J. Chiropr. Med. 15, 155–163. 10.1016/j.jcm.2016.02.012 27330520 PMC4913118

[B27] Kour N. Gupta S. Arora S. (2021). A survey of knee osteoarthritis assessment based on gait. Archives Comput. Methods Eng. 28, 345–385. 10.1007/s11831-019-09379-z

[B28] Lariviere C. Arsenault A. B. Gravel D. Gagnon D. Loisel P. (2002). 'Evaluation of measurement strategies to increase the reliability of EMG indices to assess back muscle fatigue and recovery. J. Electromyogr. Kinesiol. 12, 91–102. 10.1016/s1050-6411(02)00011-1 11955981

[B29] Lluch E. Jo N. Carol A. C. Rebbeck T. Wylde V. Baert I. (2018). Clinical descriptors for the recognition of central sensitization pain in patients with knee osteoarthritis. Disabil. rehabilitation 40, 2836–2845. 10.1080/09638288.2017.1358770 28768437

[B30] Lund J. P. Donga R. Widmer C. G. Christian S. (1991). 'The pain-adaptation model: a discussion of the relationship between chronic musculoskeletal pain and motor activity. Can. J. physiology Pharmacol. 69, 683–694. 10.1139/y91-102 1863921

[B31] Lv Z. Yang Y. X. Li J. Fei Y. Guo H. Sun Z. (2021). 'Molecular classification of knee osteoarthritis. Front. Cell Dev. Biol. 9, 725568. 10.3389/fcell.2021.725568 PMC8429960 34513847

[B32] Merletti R. Di Torino PJJEK (1999). 'Standards for reporting EMG data. J. Electromyogr. Kinesiol 9, 3–4.

[B33] Müller R. Büttner P. (1994). 'A critical discussion of intraclass correlation coefficients. Stat. Med. 13, 2465–2476. 10.1002/sim.4780132310 7701147

[B34] Norasi H. Koenig J. Mirka G. A. (2022). 'Development and assessment of a method to estimate the value of a maximum voluntary isometric contraction electromyogram from submaximal electromyographic data. J. Appl. Biomech. 38, 76–83. 10.1123/jab.2021-0229 35213822

[B35] Norcross M. F. Blackburn J. T. Goerger B. M. (2010). 'Reliability and interpretation of single leg stance and maximum voluntary isometric contraction methods of electromyography normalization. J. Electromyogr. Kinesiol 20, 420–425. 10.1016/j.jelekin.2009.08.003 19744866

[B36] O’Sullivan P. B. Grahamslaw K. M. Kendell M. Lapenskie S. C. Möller N. E. Richards K. V. (2002). The effect of different standing and sitting postures on trunk muscle activity in a pain-free population. Spine 27, 1238–1244. 10.1097/00007632-200206010-00019 12045525

[B37] Paterson K. L. Hinman R. S. Metcalf B. R. Kim L. Tim V. (2017). 'Plug-in-Gait calculation of the knee adduction moment in people with knee osteoarthritis during shod walking: comparison of two different foot marker models. J. foot ankle Res. 10, 8–9. 10.1186/s13047-017-0187-4 28174605 PMC5292150

[B38] Raez M. B. Hussain M. S. Mohd-Yasin F. (2006). 'Techniques of EMG signal analysis: detection, processing, classification and applications. Biol. Proced. Online 8, 11–35. 10.1251/bpo115 16799694 PMC1455479

[B39] Schrijvers J. C. Rutherford D. Richards R. van den Noort J. C. van der Esch M. Harlaar J. (2021). 'Inter-laboratory comparison of knee biomechanics and muscle activation patterns during gait in patients with knee osteoarthritis. Knee 29, 500–509. 10.1016/j.knee.2021.03.001 33756260

[B40] Seeley M. K. Lee H. Son S. J. Timmerman M. Lindsay M. Hopkins J. T. (2022). 'A review of the relationships between knee pain and movement neuromechanics. J. Sport Rehabil. 31, 684–693. 10.1123/jsr.2021-0020 34942599

[B41] Shiavi R. Frigo C. Pedotti A. (1998). Electromyographic signals during gait: criteria for envelope filtering and number of strides. Med. Biol. Eng. Comput. 36, 171–178. 10.1007/bf02510739 9684456

[B42] StatPearls (2023). Disclosure: ryan Siwiec declares no relevant financial relationships with ineligible companies. St. Petersburg, Florida, United States: StatPearls Publishing LLC.

[B43] Sun J. Liu Y. Yan S. Cao G. Wang S. Lester D. K. (2017). Clinical gait evaluation of patients with knee osteoarthritis. Gait Posture 58, 319–324. 10.1016/j.gaitpost.2017.08.009 28863297

[B44] Tabard-Fougère A. Rose-Dulcina K. Vincent P. Dayer R. Vuillerme N. Armand S. (2018). 'EMG normalization method based on grade 3 of manual muscle testing: within-and between-day reliability of normalization tasks and application to gait analysis. Gait posture 60, 6–12. 10.1016/j.gaitpost.2017.10.026 29121510

[B45] Thomas J. S. France C. R. Sha D. H. Vander Wiele N. (2008). The influence of pain-related fear on peak muscle activity and force generation during maximal isometric trunk exertions. Spine 33, E342–E348. 10.1097/brs.0b013e3181719264 18469681

[B46] Vigotsky A. D. Beardsley C. Contreras B. Steele J. Ogborn D. Phillips S. M. (2017). 'Greater electromyographic responses do not imply greater motor unit recruitment and 'hypertrophic potential' cannot be inferred. J. Strength Cond. Res. 31, e1–e4. 10.1519/jsc.0000000000001249 26670996

[B47] Wang K. Deng Z. Chen X. Shao J. Qiu L. Jiang C. (2023). The role of multifidus in the biomechanics of lumbar spine: a musculoskeletal modeling study. Bioeng. (Basel), 10 (1), 67. 10.3390/bioengineering10010067 PMC9854514 36671639

[B48] Warfel J. H. (1985). The extremities: muscles and motor points. New York, NY, USA: Lea & Febiger.

[B49] Watanabe H. Washio T. Saito S. Ogoh S. (2022). 'Effect of breath-hold on the responses of arterial blood pressure and cerebral blood velocity to isometric exercise. Eur. J. Appl. Physiol. 122, 157–168. 10.1007/s00421-021-04822-1 34618221

[B50] Wedege P. Steffen K. Strøm V. Arve Isak O. (2017). 'Reliability of three-dimensional kinematic gait data in adults with spinal cord injury. J. Rehabilitation Assistive Technol. Eng. 4, 10.1177/2055668317729992 PMC6453081 31186937

[B51] Yang J. F. Winter D. A. (1983). Electromyography reliability in maximal and submaximal isometric contractions. Arch. Phys. Med. Rehabil. 64 (9), 417–420. 6615179

[B52] Yocum A. McCoy S. W. Bjornson K. F. Mullens P. Gay N. (2010). 'Reliability and validity of the standing heel-rise test. Phys. Occup. Ther. Pediatr. 30, 190–204. 10.3109/01942631003761380 20608857

[B53] Zeni J. A. Richards J. G. Higginson J. S. (2008). 'Two simple methods for determining gait events during treadmill and overground walking using kinematic data. Gait Posture 27, 710–714. 10.1016/j.gaitpost.2007.07.007 17723303 PMC2384115

